# CT-based radiomics for predicting the rapid progression of coronavirus disease 2019 (COVID-19) pneumonia lesions

**DOI:** 10.1259/bjr.20201007

**Published:** 2021-04-21

**Authors:** Bin Zhang, Ma-yi-di-li Ni-jia-Ti, Ruike Yan, Nan An, Lv Chen, Shuyi Liu, Luyan Chen, Qiuying Chen, Minmin Li, Zhuozhi Chen, Jingjing You, Yuhao Dong, Zhiyuan Xiong, Shuixing Zhang

**Affiliations:** 1 Department of Radiology, The First Affiliated Hospital of Jinan University, Guangzhou, China; 2 Department of Radiology, The First People’s Hospital of Kashi area, Kashgar, China; 3 Yizhun Medical AI Co., Ltd, Beijing, China; 4 Department of Catheterization Lab, Guangdong Cardiovascular Institute, Guangdong Provincial Key Laboratory of South China Structural Heart Disease, Guangdong Provincial People’s Hospital/Guangdong Academy of Medical Sciences, Guangzhou, China; 5 Jinan University, Guangzhou, China; 6 Department of Chemical and Bio-molecular Engineering, The University of Melbourne, Melbourne, Australia

## Abstract

**Objectives:**

To develop and validate a radiomic model to predict the rapid progression (defined as volume growth of pneumonia lesions > 50% within seven days) in patients with coronavirus disease 2019 (COVID-19).

**Methods:**

Patients with laboratory-confirmed COVID-19 who underwent longitudinal chest CT between January 01 and February 18, 2020 were included. A total of 1316 radiomic features were extracted from the lung parenchyma window for each CT. The least absolute shrinkage and selection operator (LASSO), Relief, Las Vegas Wrapper (LVW), L1-norm-Support Vector Machine (L1-norm-SVM), and recursive feature elimination (RFE) were applied to select the features that associated with rapid progression. Four machine learning classifiers were used for modeling, including Support Vector Machine (SVM), Random Forest (RF), Logistic Regression (LR), and Decision Tree (DT). Accordingly, 20 radiomic models were developed on the basis of 296 CT scans and validated in 74 CT scans. Model performance was determined by the receiver operating characteristic curve.

**Results:**

A total of 107 patients (median age, 49.0 years, interquartile range, 35–54) were evaluated. The patients underwent a total of 370 chest CT scans with a median interval of 4 days (interquartile range, 3–5 days). The combination methods of L1-norm SVM and SVM with 17 radiomic features yielded the highest performance in predicting the likelihood of rapid progression of pneumonia lesions on next CT scan, with an AUC of 0.857 (95% CI: 0.766–0.947), sensitivity of 87.5%, and specificity of 70.7%.

**Conclusions:**

Our radiomic model based on longitudinal chest CT data could predict the rapid progression of pneumonia lesions, which may facilitate the CT follow-up intervals and reduce the radiation.

**Advances in knowledge:**

Radiomic features extracted from the current chest CT have potential in predicting the likelihood of rapid progression of pneumonia lesions on the next chest CT, which would improve clinical decision-making regarding timely treatment.

## Introduction

The rapid spread of coronavirus disease 2019 (COVID-19) caused by severe acute respiratory syndrome coronavirus 2 (SARS-CoV-2) as a potentially fatal disease is a major and urgent threat to global health.^1^ As of August 14, 2020, there are more than 21.05 million confirmed cases by the World Health Organization (WHO) with 752,378 deaths.^2^ Since the outbreak of COVID-19, chest CT plays an indispensable role in the detection, diagnosis, and follow-up of COVID-19 pneumonia.^3^ Chest CT not only presents the clinical course of COVID-19 infection and the disease severity but also predicts the poor outcomes of patients.^4–6^ However, multiple CT scans in short time during the COVID-19 pandemic arouses great concern about the radiation burden of the patients and healthcare workers. It is widely accepted that ionizing radiation increases the lifetime likelihood of developing cancer.^7^ Some previous studies have tried a low-dose chest CT scan in the diagnosis of COVID-19 pneumonia to reduce radiation dose.^8–12^ However, low-dose CT may miss some key signs of COVID-19 pneumonia compared with standard-dose CT. In this present study, for the first time, we aimed to develop a CT-based radiomic model to predict the probability of rapid progression of COVID-19 pneumonia to guide the follow-up interval of chest CT scan, which may reduce ionizing radiation dose and estimated cancer risk.

## Methods

### Patient data

**Figure 1.**
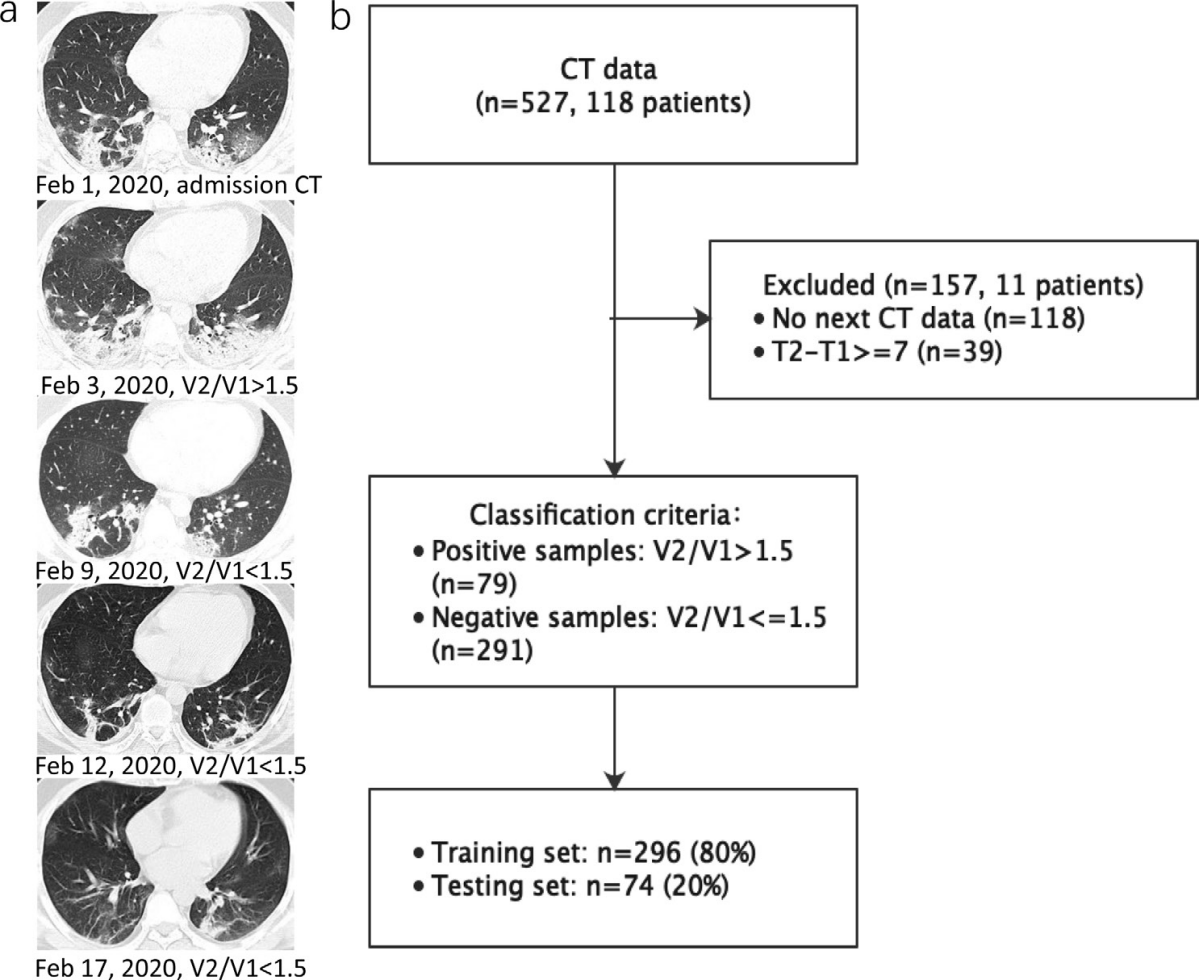
(**a**) Representative follow-up chest CT images in a male patient (29-year-old) with COVID-19 pneumonia. (**b**) Flowchart of study inclusion. Note: T2 = date of next CT examination, T1 = date of this CT examination, V2 = the pneumonia volume on the next CT examination, V1 = the pneumonia volume on this CT examination.

**Figure 2.**
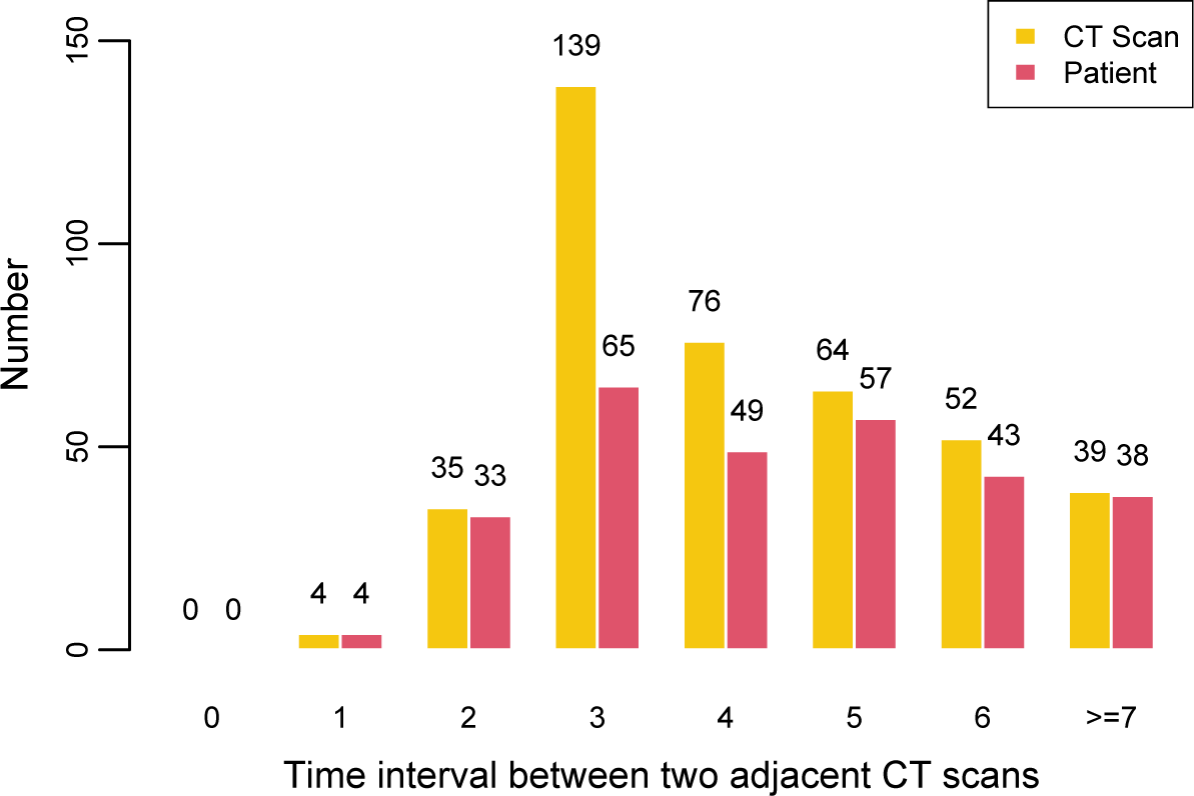
Histogram of the distribution of number of CT scans and patients under different time interval between two adjacent CT scans.

This study was approved by the institutional review board and the need for written informed consent was waived. A total of 118 COVID-19 patients from two designated hospitals were retrospectively included between January 8, 2020 and February 25, 2020. Adult patients had a laboratory-confirmed COVID-19, which was achieved by real-time reverse transcription-polymerase chain reaction (RT-PCR) assay of throat swab samples (at least two samples were taken, at least 24 h apart) for COVID-19 according to the protocol established by the WHO. The 118 CT scans from 11 patients were excluded due to no follow-up CT scans or the interval time of two adjacent CT scans >7 days. Finally, a total of 370 CT scans from 107 patients were analyzed in this study. The whole dataset was randomly divided into two subsets, 80% for training and the remaining 20% for validation using 10-fold cross-validation. A representative case and the flowchart of patients and CT scans inclusion are shown in Figure 1. The distribution of number of CT scans and patients under different time interval between two adjacent CT scans is illustruted in Figure 2.

### CT image acquisitions

Patients underwent chest CT scans by CT 64 scanner (GE Medical System), Siemens Emotion 16 scanner (Siemens Healthineers; Erlangen, Germany), or ICT 128 scanner (Philips Healthcare, Netherlands). No contrast agent was administered. CT acquisition parameters of the three CT scanners were shown in Table 1.

**Table 1.** The parameters of CT image acquisitions

CT scanner	Manufacturer	Tube voltage	Tube current	Pitch	Slice Thickness
64 scanner	GE	120 kV	260 mA	0.984	0.625 mm
16 scanner	Siemens	130 kV	automatic	1.5	1.0 or 0.6 mm
128 scanner	Philips	120 kV	automatic	0.7	1.0 or 0.67 mm

### CT image segmentation

We used a previously trained 3D U-net ++that based on 2000 COVID-19 pneumonia cases to pre-segment the COVID-19 pneumonia lesions in this present study. Several days later, an experienced radiologist (with more than 15 years’ experience in chest imaging) edited and verified the pre-segmentation results, removed false positives and segmented the missed lesions.

### Radiomic feature extraction

All raw CT images were preprocessed by 1 mm*1 mm*1 mm resampling. Radiomic features were extracted from the lung window (window width: 1500 Hounsfield Unit [HU], window level: 600 HU) in the Python (v.3.7.0, Beaverton, Ore; https://www.python.org/) by using the Pyradiomics package (v.3.0; https://github.com/Radiomics/pyradiomics). The parameters used in different transforms in c were presented in Table 2. A total of 1316 radiomic features were extracted under seven image types including Originial, Wavelet, LoG, Square, SquareRoot, Exponential and Logarithm. The class and corresponding number of radiomics features are presented in Table 3. All radiomic features were normalized by min_max.

**Table 2.** The parameters used in different transforms in Pyradiomics

Transform	Original	Wavelet	LoG	Square	SquareRoot	Exponential	Logarithm
Parameters	default	default	sigma = 3.0	default	default	default	default

**Table 3.** The number of radiomics features in different categories under different image types

Image type Feature type	Original	Wavelet	LoG	Square	SquareRoot	Exponential	Logarithm
Shape-based features	14	–	–	–	–	–	–
First-order statistics	18	144	18	18	18	18	18
GLCM	24	192	24	24	24	24	24
GLDM	14	112	14	14	14	14	14
GLRLM	16	128	16	16	16	16	16
GLSZM	16	128	16	16	16	16	16
NGTDM	5	40	5	5	5	5	5

GLCM, Gray-level co-occurrence matrix; GLDM, Gray-level dependence matrix; GLRLM, Gray-level run length matrix; GLSZM, Gray-level size zone matrix; NGTDM, Neighboring gray tone difference matrix.

### Radiomic feature selection

Considering the high-dimensional radiomic features may contain redundant information, five feature selectors including the least absolute shrinkage and selection operator (LASSO),^13^ Relief,^14^ Las Vegas Wrapper (LVW),^15^ L1-norm-Support Vector Machine (L1-norm-SVM),^16^ and recursive feature elimination (RFE)^17^ were used to reduce the dimensions of the features before the machine learning was applied to train the models.

### Machine-learning-based radiomic model construction

Rapid progression of pneumonia lesions meant volume growth >50% within seven days, which was calculated as the ratio of the pneumonia volume on the next CT scan (V2) to the pneumonia volume on the current CT scan (V1). The threshold of 50% was identified according to the COVID-19 guidelines (trial version 6) released by the National Health Commission of China.^18^ V2/V1 >1.5 indicates that the current CT scan is a positive sample, otherwise, a negative sample. For unbiased estimates of diagnostic accuracy, our dataset was randomly split into training and validation datasets with a ratio of 4:1. The proportions of positive and negative samples in training and testing datasets were the same when splitting the dataset. Four common machine learning algorithms including Support Vector Machine (SVM), Random Forest (RF), Logistic Regression (LR) and Decision Tree (DT) were applied to predict the occurrence of rapid progression. To select the optimal model and hyperparameters for each model, we conducted 10-fold cross-validation on each training dataset. The hyperparameters of 20 combination models were showed in Supplementary Material 1. The model with the highest area under the curve (AUC) is considered to be the optimal model. All models building was performed in the Python environment (v.3.7.0, https://www.python.org/) by using the Scikit-learn package (v.0.23.1; https://scikit-learn.org/).

Supplementary Material 1.

### Statistical analysis

Categorical variables were expressed as counts and percentage, while continuous variables are shown as median and interquartile range. All the statistical analyses were performed using Python, v.3.7.0 (Beaverton, Ore; https://www.python. org/). The packages were used as follows: “mlr” for LR, “randomForest” for RF, and “e1071” for SVM. Receiver operating characteristic curve (ROC) analyses were performed to evaluate the performance of different models to predict the occurrence of rapid progression. The AUC comparison of different models used Delong test.^19^ A *p* < 0.05 was considered significant.

## Results

A total of 107 patients with COVID-19 were included in this study, with median age of 49.0 years (interquartile range, 35–54) and 60 (56.6%) were males. 11 (10.3%) had hypertension, 5 (4.7%) had diabetes, 3 (2.8%) had coronary heart disease, 2 (1.9%) had chronic liver disease, 2 (1.9%) had chronic lung disease, and 5 (4.7%) had previous surgery. The patients underwent a total of 370 chest CT scans with a median interval of 4 days (interquartile range, 3–5 days). The median time from the initial to the last CT scan was 15 days (interquartile range, 12–22).

**Figure 3.**
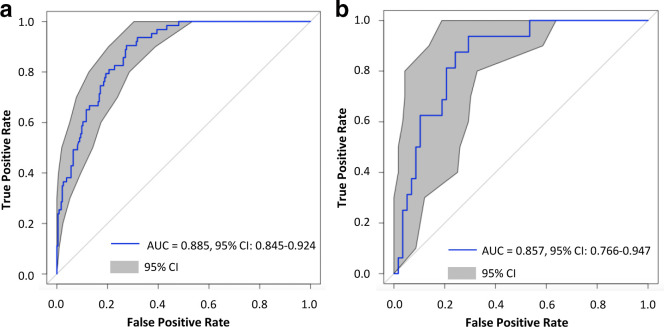
The ROC curves of the training dataset and testing dataset with the optimal model. The left panel shows the mean ROC curve and the 95% CI for the training dataset (a). The right panel shows the mean ROC curve and the 95% CI for the validation dataset (b).

After selection by LASSO, Relief, LVW, L1-norm SVM, and RFE, 11, 18, 2–24, 17, and 44 features were identified respectively. Among the 20 combined models, L1-norm SVM +SVM with 17 radiomic features achieved the optimal predictive performance, with an AUC of 0.885 (95% CI: 0.845–0.924), sensitivity of 93.7%, specificity of 63.5%, and accuracy of 69.9% in the training dataset and an AUC of 0.857 (95% CI: 0.766–0.947), sensitivity of 87.5%, specificity of 70.7%, and accuracy of 74.3% in the internal-validation dataset (Figure 3, Table 4) The AUC of L1-norm SVM +SVM was higher than that of all classifiers with the exception of LASSO + SVM (0.844), Relief + LR (0.842), L1-norm SVM +LR (0.851), and RFE + LR (0.834) (Delong test: *p* = 0.402, 0.218, 0.725, and 0.271, respectively (Supplementary Table 1). The radiomic risk signature based on three first-order statistics and 14 texture features is illustrated as follows:

Supplementary Table 1.

Radiomic risk signature =

**Table 4.** The performance of 20 combinations of machine learning methods in predicting the occurrence of rapid progression in patients with COVID-19

Model	No. of features	Train_ ACC	Train_ SN	Train_ SP	Train_ PPV	Train_ NPV	Train_AUC (95% CI)	Test_ ACC	Test_ SN	Test_ SP	Test_ PPV	Test_ NPV	Test_AUC (95% CI)
LASSO + SVM	11	69.6%	92.1%	63.5%	40.6%	96.7%	0.854 (0.809–0.898)	71.6%	87.5%	67.2%	42.4%	95.1%	0.844 (0.750–0.938)
LASSO + LR	11	75.7%	81.0%	74.3%	46.0%	93.5%	0.860 (0.817–0.904)	77.0%	62.5%	81.0%	47.6%	88.7%	0.825 (0.724–0.927)
LASSO + DT	11	98.7%	100.0%	98.3%	94.0%	100.0%	1.000 (0.999–1.000)	75.7%	43.8%	84.5%	43.8%	84.5%	0.645 (0.510–0.780)
LASSO + RF	11	97.0%	87.3%	99.6%	98.2%	96.7%	0.998 (0.996–1.000)	75.7%	0	96.6%	0	77.8%	0.736 (0.611–0.860)
Relief + SVM	18	65.2%	73.0%	63.1%	34.9%	89.6%	0.777 (0.717–0.837)	71.6%	81.3%	69.0%	41.9%	93.0%	0.802 (0.701–0.903)
Relief + LR	18	73.0%	81.0%	70.8%	42.9%	93.2%	0.831 (0.779–0.883)	77.0%	68.8%	79.3%	47.8%	90.2%	0.842 (0.749–0.935)
Relief + DT	18	100.0%	100.0%	100.0%	100.0%	100.0%	1.000 (1.000–1.000)	73.0%	12.5%	89.7%	25.0%	78.8%	0.511 (0.418–0.603)
Relief + RF	18	90.9%	60.3%	99.1%	95.0%	90.2%	0.920 (0.878–0.962)	81.1%	12.5%	100.0%	100.0%	80.6%	0.713 (0.577–0.850)
LVW + SVM	2	60.1%	74.6%	56.2%	31.5%	89.1%	0.758 (0.696–0.820)	54.1%	50.0%	55.2%	23.5%	80.0%	0.642 (0.492–0.792)
LVW + LR	5	68.2%	77.8%	65.7%	38.0%	91.6%	0.789 (0.732–0.845)	71.6%	75.0%	70.7%	41.4%	91.1%	0.780 (0.669–0.892)
LVW + DT	24	99.0%	100.0%	98.7%	95.5%	100.0%	0.999 (0.997–1.000)	68.9%	31.3%	79.3%	29.4%	80.7%	0.554 (0.424–0.685)
LVW + RF	6	98.0%	90.5%	100.0%	100.0%	97.5%	0.999 (0.997–1.000)	77.0%	12.5%	94.8%	40.0%	79.7%	0.671 (0.524–0.818)
L1-norm-SVM+SVM	17	69.9%	93.7%	63.5%	41.0%	97.4%	0.885 (0.845–0.924)	74.3%	87.5%	70.7%	45.2%	95.4%	0.857 (0.766–0.947)
L1-norm-SVM+LR	17	77.7%	84.1%	76.0%	48.6%	94.7%	0.889 (0.851–0.927)	81.1%	75.0%	82.8%	54.6%	92.3%	0.851 (0.753–0.949)
L1-norm-SVM+DT	17	100.0%	100.0%	100.0%	100.0%	100.0%	1.000 (1.000–1.000)	81.1%	31.3%	94.8%	62.5%	83.3%	0.630 (0.510–0.751)
L1-norm-SVM+RF	17	98.7%	93.7%	100.0%	100.0%	98.3%	1.000 (0.999–1.000)	81.1%	31.3%	94.8%	62.5%	83.3%	0.728 (0.581–0.876)
RFE + SVM	44	88.2%	100.0%	85.0%	64.3%	100.0%	0.954 (0.932–0.976)	73.0%	62.5%	75.9%	41.7%	88.0%	0.776 (0.666–0.875)
RFE + LR	44	83.1%	92.1%	80.7%	56.3%	97.4%	0.930 (0.901–0.960)	77.0%	62.5%	81.0%	47.6%	88.7%	0.834 (0.738–0.931)
RFE + DT	44	100.0%	100.0%	100.0%	100.0%	100.0%	1.000 (1.000–1.000)	70.3%	18.8%	84.5%	25.0%	79.0%	0.516 (0.407–0.626)
RFE + RF	44	97.3%	87.3%	100.0%	100.0%	96.7%	0.998 (0.996–1.000)	78.4%	6.3%	98.3%	50.0%	79.2%	0.658 (0.506–0.809)

ACC, Accuracy; AUC, Area under the curve; DT, Decision tree;LASSO, Least absolute shrinkage and selection operator; LR, Logistic regression; LVW, Las vegas wrapper; NPV, Negative predictive value; PPV, Positive predictive value; RF, Random forest;SP, Specificity; SVM, Support vector machine.

4.35*original_glcm_Imc2

+1.56 *wavelet_LHL_firstorder_TotalEnergy

+1.58 *wavelet_LHL_gldm_LargeDependenceLowGrayLevelEmphasis

+8.42 *wavelet_LHL_glrlm_GrayLevelNonUniformityNormalized

−1.25*wavelet_LHL_glrlm_RunEntropy

+1.81 *wavelet_LHL_glszm_GrayLevelNonUniformityNormalized

−8.60*wavelet_LHH_glszm_ZoneVariance

−1.30*wavelet_HHL_glrlm_LongRunHighGrayLevelEmphasis

+2.46*wavelet_HHH_firstorder_Kurtosis

+0.82*wavelet_HHH_gldm_DependenceEntropy

−2.01*wavelet_HHH_glszm_GrayLevelNonUniformityNormalized

+3.54*wavelet_LLL_glszm_GrayLevelVariance

−8.49*square_glcm_JointEnergy

−6.82*squareroot_glcm_Imc2

−1.07*exponential_glrlm_RunVariance

+2.92 logarithm_firstorder_InterquartileRange

−0.65*logarithm_glszm_SmallAreaLowGrayLevelEmphasis

−1.72

## Discussion

In this present study, we developed and validated radiomic models based on this chest CT scan to predict the probability of rapid progression of COVID-19 pneumonia on next CT scan. We combined five feature selection methods and four classification methods and the results showed that the combination of L1-norm SVM +SVM outperformed other combinations, yielding an AUC of 0.857 (95% CI: 0.766–0.947), sensitivity of 87.5%, specificity of 70.7%, and accuracy of 74.3%.

Radiomics is a quantitative tool for medical imaging, which enhances the existing data available to clinicians by means of advanced mathematical analysis from the field of machine learning. This novel approach has been widely used in diagnosing, staging, predicting treatment response and prognosis of cancers. Since the outbreak of COVID-19, some studies have applied CT-based radiomics to copy with this emergent infectious diseases. By high-throughput extracting huge amounts of features from chest CT images of COVID-19 pneumonia, radiomics can reflect underlying information that associates with disease heterogeneity. Fang et al developed a radiomic nomogram with high performance in differentiating COVID-19 from other types of viral pneumonia.^20^ Recent studies also used radiomics to diagnose and predict the outcomes of COVID-19. For example, researchers proposed a non-invasive and quantitative radiomic model using CT to predict poor outcomes in advance among COVID-19 patients.^21–23^ Wei et al^24^ and Tang et al^25^ found that CT texture features could distinguish non-severe and severe COVID-19 patients. Fu et al^26^ used radiomics and machine learning to predict the progressive from stable COVID-19 infection in the early stage. Yue et al^27^ developed and tested machine learning-based CT radiomics models for predicting hospital stay in patients with COVID-19 pneumonia. Cai et al^28^ built a model based on chest CT radiomic features and clinical characteristics to predict RT-PCR turning negative during clinical treatment. Current evidence demonstrated that radiomics has potential in the clinical management of COVID-19. Unlike previous studies, this study provided a radiomic tool to predict COVID-19 patients who had high-risk of rapid progression of pneumonia within seven days and the results showed promising.

The performance of a radiomic model would be affected by the each step of radiomic workflow. The differences in noise and resolution of CT images from the different CT systems may impact the reproducibility of radiomics, for instance, different values of radiomic features.^29^ We used image pre-processing to reduce the bias caused by different scanners and imaging protocols. According to previous studies, we extracted three common classes of radiomic features (first-order statistics, shape-based features and texture features) by using the Pyradiomics package. Regarding the various choices of feature selection and modelling methodologies, the identification of optimal machine learning methods for radiomic applications is a crucial step towards stable and clinically relevant clinical-decision support systems; thus, multiple machine-learning methods should be employed and compared. In this study, we chose five feature selectors and four modeling methods to identify the best combination and found that L1-norm SVM +SVM achieved the highest performance in the specific task of predicting rapid progression of COVID-19. Zhang et al^30^ evaluated six feature selection methods and nine classifiers to predict the recurrence and distant metastasis in patients with advanced nasopharyngeal carcinoma and found that the combination methods Random Forest (RF) +RF performed the best. In this study, we identified 17 radiomic features that were most strongly related to the prediction outcome, consisting of 3 first-order statistics and 14 texture features. All are associated with image uniformity and heterogeneity. COVID-19 pneumonia lesions with high-risk of rapid progression were more heterogeneous (*e.g.* mixed ground-glass opacity and consolidation) than those with low-risk of rapid progression. Previous studies have showed that radiomics or texture analysis can characterize tumor phenotypes and reflect the tumor heterogeneity.^31,32^ This study also demonstrated that radiomics features can serve as an effective biomarker of COVID-19 pneumonia by reflecting the heterogeneity of lesions.

This study also has some limitations. First, the retrospective nature of this study. Second, the clinical and laboratory variables did not be integrated into the prediction model because they were not matched with the each chest CT examinations. Third, the effect of treatment on the COVID-19 pneumonia did not be considered because there were no specific treatment of COVID-19. The drugs of COVID-19 used in clinical setting were mixed although there were recommends of guidelines. Finally, this model lacks of external validation, whose generalization needs to be tested in other institutions.

In conclusion, we proposed a CT-based radiomic model to predict the rapid progression of COVID-19 pneumonia, which may rationalize the chest CT follow-up intervals of COVID-19 patients and would benefit the clinical management of COVID-19 patients.
