# Supplementary material for: CT-based radiomics for predicting the rapid progression of coronavirus disease 2019 (COVID-19) pneumonia lesions
Source: Br J Radiol. 2021 Apr 21;94(1122):20201007. doi: 10.1259/bjr.20201007 (PMC8173680; doi:10.1259/bjr.20201007)
Supplement: Supplementary Material 1. [file bjr.20201007.suppl-02.docx]

**Supplementary Material**

Optimal hyperparameters of SVM classifier based on LASSO feature selection method were as follows.

- kernel: rbf
- class_weight: balanced
- regularization parameter C: 0.1
- gamma: 1.25

Optimal hyperparameters of LR classifier based on LASSO feature selection method were as follows.

- penalty: l2
- class_weight: balanced
- max_iter: 20

Optimal hyperparameters of DT classifier based on LASSO feature selection method were as follows.

- criterion: gini
- max_depth: 12
- min_samples_split: 2
- min_samples_leaf: 1
- max_features: auto
- class_weight: balanced

Optimal hyperparameters of RF classifier based on LASSO feature selection method were as follows.

- criterion: gini
- max_depth: None
- min_samples_split: 2
- min_samples_leaf: 1
- max_features: auto
- class_weight: balanced
- n_estimators: 8

Optimal hyperparameters of SVM classifier based on Relief feature selection method were as follows.

- kernel: rbf
- class_weight: balanced
- regularization parameter C: 0.05
- gamma: 0.3125

Optimal hyperparameters of LR classifier based on Relief feature selection method were as follows.

- penalty: l2
- class_weight: balanced
- max_iter: 20

Optimal hyperparameters of DT classifier based on Relief feature selection method were as follows.

- criterion: gini
- max_depth: 12
- min_samples_split: 2
- min_samples_leaf: 1
- max_features: auto
- class_weight: balanced

Optimal hyperparameters of RF classifier based on Relief feature selection method were as follows.

- criterion: gini
- max_depth: None
- min_samples_split: 2
- min_samples_leaf: 1
- max_features: auto
- class_weight: balanced
- n_estimators: 2

Optimal hyperparameters of SVM classifier based on LVW feature selection method were as follows.

- kernel: rbf
- class_weight: balanced
- regularization parameter C: 0.1
- gamma: 10.0

Optimal hyperparameters of LR classifier based on LVW feature selection method were as follows.

- penalty: l2
- class_weight: balanced
- max_iter: 20

Optimal hyperparameters of DT classifier based on LVW feature selection method were as follows.

- criterion: gini
- max_depth: 10
- min_samples_split: 2
- min_samples_leaf: 1
- max_features: auto
- class_weight: balanced

Optimal hyperparameters of RF classifier based on LVW feature selection method were as follows.

- criterion: gini
- max_depth: None
- min_samples_split: 2
- min_samples_leaf: 1
- max_features: auto
- class_weight: balanced
- n_estimators: 10

Optimal hyperparameters of SVM classifier based on L1-norm-SVM feature selection method were as follows.

- kernel: rbf
- class_weight: balanced
- regularization parameter C: 0.1
- gamma: 0.625

Optimal hyperparameters of LR classifier based on L1-norm-SVM feature selection method were as follows.

- penalty: l2
- class_weight: balanced
- max_iter: 20

Optimal hyperparameters of DT classifier based on L1-norm-SVM feature selection method were as follows.

- criterion: gini
- max_depth: 14
- min_samples_split: 2
- min_samples_leaf: 1
- max_features: auto
- class_weight: balanced

Optimal hyperparameters of RF classifier based on L1-norm-SVM feature selection method were as follows.

- criterion: gini
- max_depth: None
- min_samples_split: 2
- min_samples_leaf: 1
- max_features: auto
- class_weight: balanced
- n_estimators: 10

Optimal hyperparameters of SVM classifier based on RFE feature selection method were as follows.

- kernel: rbf
- class_weight: balanced
- regularization parameter C: 0.1
- gamma: 2.5

Optimal hyperparameters of LR classifier based on RFE feature selection method were as follows.

- penalty: l2
- class_weight: balanced
- max_iter: 20

Optimal hyperparameters of DT classifier based on RFE feature selection method were as follows.

- criterion: gini
- max_depth: 14
- min_samples_split: 2
- min_samples_leaf: 1
- max_features: auto
- class_weight: balanced

Optimal hyperparameters of RF classifier based on RFE feature selection method were as follows.

- criterion: gini
- max_depth: None
- min_samples_split: 2
- min_samples_leaf: 1
- max_features: auto
- class_weight: balanced
- n_estimators: 6
