# Supplementary material for: CT-based radiomics for predicting the rapid progression of coronavirus disease 2019 (COVID-19) pneumonia lesions
Source: Br J Radiol. 2021 Apr 21;94(1122):20201007. doi: 10.1259/bjr.20201007 (PMC8173680; doi:10.1259/bjr.20201007)
Supplement: Supplementary Table 1. [file bjr.20201007.suppl-01.docx]

**Abbreviations:** SVM, Support Vector Machine; LASSO, Least Absolute Shrinkage and Selection Operator; LR, Logistic Regression; DT, Decision Tree; RF, Random Forest; LVW, Las Vegas Wrapper; RFE, Recursive Feature Elimination.

Supplementary Table 1. Comparisons of receiver operating characteristic curves between prediction models

| Model | 1 | 2 | 3 | 4 | 5 | 6 | 7 | 8 | 9 | 10 | 11 | 12 | 13 | 14 | 15 | 16 | 17 | 18 | 19 | 20 |
| --- | --- | --- | --- | --- | --- | --- | --- | --- | --- | --- | --- | --- | --- | --- | --- | --- | --- | --- | --- | --- |
| 1 | 1.0000 | 0.1798 | 0.0000 | 0.0000 | 0.0042 | 0.8838 | 0.0000 | 0.0036 | 0.0000 | 0.0002 | 0.0000 | 0.0000 | 0.4016 | 0.6467 | 0.0000 | 0.0023 | 0.0001 | 0.3970 | 0.0000 | 0.0000 |
| 2 | 0.1798 | 1.0000 | 0.0002 | 0.0000 | 0.1891 | 0.3340 | 0.0000 | 0.0135 | 0.0000 | 0.0033 | 0.0000 | 0.0000 | 0.0465 | 0.1052 | 0.0002 | 0.0061 | 0.0029 | 0.5749 | 0.0000 | 0.0000 |
| 3 | 0.0000 | 0.0002 | 1.0000 | 0.0602 | 0.0009 | 0.0001 | 0.0306 | 0.1096 | 0.9478 | 0.0052 | 0.0455 | 0.6063 | 0.0001 | 0.0001 | 0.7578 | 0.0948 | 0.0061 | 0.0000 | 0.0233 | 0.8088 |
| 4 | 0.0000 | 0.0000 | 0.0602 | 1.0000 | 0.0097 | 0.0001 | 0.0001 | 0.6437 | 0.0074 | 0.0251 | 0.0002 | 0.0229 | 0.0000 | 0.0000 | 0.0484 | 0.8451 | 0.0653 | 0.0001 | 0.0001 | 0.0331 |
| 5 | 0.0042 | 0.1891 | 0.0009 | 0.0097 | 1.0000 | 0.0042 | 0.0000 | 0.0724 | 0.0004 | 0.1507 | 0.0000 | 0.0003 | 0.0041 | 0.0224 | 0.0011 | 0.0870 | 0.0326 | 0.0391 | 0.0000 | 0.0015 |
| 6 | 0.8838 | 0.3340 | 0.0001 | 0.0001 | 0.0042 | 1.0000 | 0.0000 | 0.0098 | 0.0001 | 0.0001 | 0.0000 | 0.0000 | 0.2184 | 0.6182 | 0.0000 | 0.0130 | 0.0005 | 0.7045 | 0.0000 | 0.0002 |
| 7 | 0.0000 | 0.0000 | 0.0306 | 0.0001 | 0.0000 | 0.0000 | 1.0000 | 0.0014 | 0.0492 | 0.0000 | 0.4086 | 0.0156 | 0.0000 | 0.0000 | 0.0227 | 0.0019 | 0.0000 | 0.0000 | 0.8760 | 0.0315 |
| 8 | 0.0036 | 0.0135 | 0.1096 | 0.6437 | 0.0724 | 0.0098 | 0.0014 | 1.0000 | 0.1409 | 0.1751 | 0.0048 | 0.2954 | 0.0040 | 0.0060 | 0.1503 | 0.6968 | 0.1832 | 0.0038 | 0.0012 | 0.1314 |
| 9 | 0.0000 | 0.0000 | 0.9478 | 0.0074 | 0.0004 | 0.0001 | 0.0492 | 0.1409 | 1.0000 | 0.0006 | 0.0571 | 0.4295 | 0.0000 | 0.0000 | 0.8101 | 0.0293 | 0.0006 | 0.0000 | 0.0388 | 0.6927 |
| 10 | 0.0002 | 0.0033 | 0.0052 | 0.0251 | 0.1507 | 0.0001 | 0.0000 | 0.1751 | 0.0006 | 1.0000 | 0.0000 | 0.0003 | 0.0002 | 0.0010 | 0.0030 | 0.1687 | 0.7537 | 0.0026 | 0.0000 | 0.0042 |
| 11 | 0.0000 | 0.0000 | 0.0455 | 0.0002 | 0.0000 | 0.0000 | 0.4086 | 0.0048 | 0.0571 | 0.0000 | 1.0000 | 0.0325 | 0.0000 | 0.0000 | 0.0015 | 0.0039 | 0.0000 | 0.0000 | 0.3605 | 0.0840 |
| 12 | 0.0000 | 0.0000 | 0.6063 | 0.0229 | 0.0003 | 0.0000 | 0.0156 | 0.2954 | 0.4295 | 0.0003 | 0.0325 | 1.0000 | 0.0000 | 0.0000 | 0.4962 | 0.0074 | 0.0009 | 0.0000 | 0.0132 | 0.6998 |
| 13 | 0.4016 | 0.0465 | 0.0001 | 0.0000 | 0.0041 | 0.2184 | 0.0000 | 0.0040 | 0.0000 | 0.0002 | 0.0000 | 0.0000 | 1.0000 | 0.7249 | 0.0000 | 0.0035 | 0.0005 | 0.2712 | 0.0000 | 0.0000 |
| 14 | 0.6467 | 0.1052 | 0.0001 | 0.0000 | 0.0224 | 0.6182 | 0.0000 | 0.0060 | 0.0000 | 0.0010 | 0.0000 | 0.0000 | 0.7249 | 1.0000 | 0.0001 | 0.0012 | 0.0013 | 0.4174 | 0.0000 | 0.0000 |
| 15 | 0.0000 | 0.0002 | 0.7578 | 0.0484 | 0.0011 | 0.0000 | 0.0227 | 0.1503 | 0.8101 | 0.0030 | 0.0015 | 0.4962 | 0.0000 | 0.0001 | 1.0000 | 0.1124 | 0.0049 | 0.0001 | 0.0156 | 0.6651 |
| 16 | 0.0023 | 0.0061 | 0.0948 | 0.8451 | 0.0870 | 0.0130 | 0.0019 | 0.6968 | 0.0293 | 0.1687 | 0.0039 | 0.0074 | 0.0035 | 0.0012 | 0.1124 | 1.0000 | 0.1938 | 0.0026 | 0.0017 | 0.0172 |
| 17 | 0.0001 | 0.0029 | 0.0061 | 0.0653 | 0.0326 | 0.0005 | 0.0000 | 0.1832 | 0.0006 | 0.7537 | 0.0000 | 0.0009 | 0.0005 | 0.0013 | 0.0049 | 0.1938 | 1.0000 | 0.0004 | 0.0000 | 0.0018 |
| 18 | 0.3970 | 0.5749 | 0.0000 | 0.0001 | 0.0391 | 0.7045 | 0.0000 | 0.0038 | 0.0000 | 0.0026 | 0.0000 | 0.0000 | 0.2712 | 0.4174 | 0.0001 | 0.0026 | 0.0004 | 1.0000 | 0.0000 | 0.0000 |
| 19 | 0.0000 | 0.0000 | 0.0233 | 0.0001 | 0.0000 | 0.0000 | 0.8760 | 0.0012 | 0.0388 | 0.0000 | 0.3605 | 0.0132 | 0.0000 | 0.0000 | 0.0156 | 0.0017 | 0.0000 | 0.0000 | 1.0000 | 0.0269 |
| 20 | 0.0000 | 0.0000 | 0.8088 | 0.0331 | 0.0015 | 0.0002 | 0.0315 | 0.1314 | 0.6927 | 0.0042 | 0.0840 | 0.6998 | 0.0000 | 0.0000 | 0.6651 | 0.0172 | 0.0018 | 0.0000 | 0.0269 | 1.0000 |

**Notes:** Model 1, LASSO + SVM; Model 2, LASSO + LR; Model 3, LASSO + DT; Model 4, LASSO + RF; Model 5, Relief + SVM; Model 6, Relief + LR; Model 7, Relief + DT; Model 8, Relief + RF; Model 9, LVW + SVM; Model 10, LVW + LR; Model 11, LVW + DT; Model 12, LVW + RF; Model 13, L1-norm-SVM + SVM; Model 14, L1-norm-SVM + LR; Model 15, L1-norm-SVM + DT; Model 16, L1-norm-SVM + RF; Model 17, RFE + SVM; Model 18, RFE + LR; Model 19, RFE + DT; Model 20, RFE + RF.
